# The Sleeping Brain's Influence on Verbal Memory: Boosting Resistance to Interference

**DOI:** 10.1371/journal.pone.0004117

**Published:** 2009-01-07

**Authors:** Jeffrey M. Ellenbogen, Justin C. Hulbert, Ying Jiang, Robert Stickgold

**Affiliations:** 1 Department of Neurology, Massachusetts General Hospital, Harvard Medical School, Boston, Massachusetts, United States of America; 2 University of St. Andrews, St. Andrews, United Kingdom; 3 Dartmouth College, Hanover, United States of America; 4 Beth Israel Deaconess Medical Center, Harvard Medical School, Boston, Massachusetts, United States of America; University of Sydney, Australia

## Abstract

Memories evolve. After learning something new, the brain initiates a complex set of post-learning processing that facilitates recall (i.e., consolidation). Evidence points to sleep as one of the determinants of that change. But whenever a behavioral study of episodic memory shows a benefit of sleep, critics assert that sleep only leads to a temporary shelter from the damaging effects of interference that would otherwise accrue during wakefulness. To evaluate the potentially active role of sleep for verbal memory, we compared memory recall after sleep, with and without interference before testing. We demonstrated that recall performance for verbal memory was greater after sleep than after wakefulness. And when using interference testing, that difference was even more pronounced. By introducing interference after sleep, this study confirms an experimental paradigm that demonstrates the active role of sleep in consolidating memory, and unmasks the large magnitude of that benefit.

## Introduction

In decades past, to mention sleep and memory in the same context was to conjure up notions of sleep–thought to be a state of neurobiological quiescence–as providing a respite from memory loss in the day (i.e., sleep would provide a *transient and passive* shelter from the damaging effects of interference that would otherwise take place while awake). This perspective was undoubtedly inherited through its originators, Jenkins and Dallenbach [1], who, in their groundbreaking work on sleep and memory, demonstrated greater recall after sleep than after similar periods of time awake. Rather than ascribe a benefit of sleep, however, the authors concluded from their study that the wake group was performing worse. They reasoned that the wake participants were exposed to new information that compromised the previously learned information (i.e., interference). In short, sleep provided no meaningful contribution to memory.

At the time of their study, the prevailing (albeit inaccurate) account of sleep was that it was a state of “diffuse cortical inhibition” [2]. So it made more sense to focus on the negative effects of waking experience on memory, rather than attribute some biological phenomenon in sleep that actively strengthened memory.

In recent years, however, a multitude of studies have demonstrated the complex neurobiology of sleep. It is now known, for example, that all stages of sleep have some form of cerebral activity: Among these, there are active brain regions during REM sleep [3] and spontaneous firing of collections of neurons in non-REM sleep [4].

A growing body of converging evidence points to sleep as contributing to memory consolidation, in particular. Animal studies, computational models, neuroimaging studies, electrophysiology and behavioral experiments support the notion that a memory undergoes a process of transition after learning, and that process is influenced by sleep [for reviews, see 5], [6], [7], [8], [9].

Behavioral evidence—that sleep enhances episodic memory consolidation—derives largely from experiments that test memory recall after periods of sleep, compared to similar periods of time awake. While important insights are learned from this body of literature, an essential question remains: do these data show that sleep actively contributes to memory, or do they merely demonstrate that sleep transiently shelters memory from the damaging effects of interference that occur in the waking state?

Hence a scientific stalemate. That is, even if data show a sleep group performing better than a wake (control) group, two equally compelling conclusions can be drawn: either the sleep group is performing better, or, the wake group is performing worse. Looking at the very same data, some will claim that sleep is making a meaningful contribution to memory, while others point to a phenomenon of interference as the culprit that artificially inflates the sleep group, simply by making the wake group perform worse.

Fortunately, this disagreement forms the basis of a testable hypothesis: if sleep merely prevents interference, providing a temporary respite for newly formed memories, then, after sleeping, those memories would be vulnerable to interference once again. If, however, sleep helps consolidate memories (for example, through its generation of synchronous neural oscillations, or through shifts in neuromodulator levels, or blockade of sensory input) then, after sleeping, those memories should be more resistant to interference. In a recent study [10] sleep was found to consolidate memories, stabilizing them such that they became resistant to the effects of interference in the subsequent day. The current study seeks to replicate that novel finding in a different population and with a refined behavioral paradigm.

## Methods

**Figure 1:**
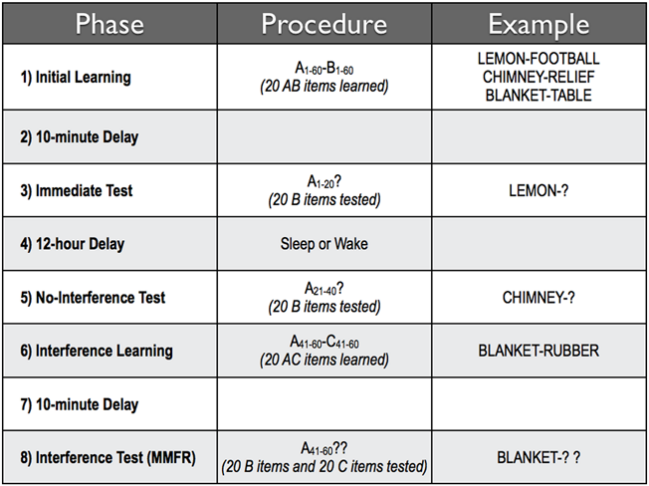
Design. Initially, all participants studied 60 pairs of words, schematically represented as A–B (Lemon-Football, Chimney-Relief, Blanket-Table, etc.). Ten minutes after training, 20 of those word pairs were selected for testing (e.g., Lemon-?). Then a different 20 pairs, from the 60 learned, were tested after a 12-hour delay containing either sleep or wakefulness (e.g., Chimney-?). Participants next learned twenty A–C pairs (e.g., Blanket-Rubber), each of which shared a cue-word (A-) with a member of one of the 20 pairs that was not yet tested. This training has been shown to induce retroactive interference for the A–B pairs, but was predicted to be less disruptive if the earlier memories had been consolidated during sleep. Ten minutes after learning the A–C list, participants were provided with the third set of A-cues and asked to recall *both* the original response (B-items; e.g., Table) as well as the new responses (C-items; e.g., Rubber) on the Modified-Modified Free Recall (MMFR) test. Testing lists of 20 items were counterbalanced across participants.

To achieve this experimental manipulation, we employed an A–B, A–C interfering word-pair paradigm [11], using the amended modified, modified free recall (MMFR) procedure [12]. [For a discussion of this paradigm, see 13]. In our adaptation, we experimentally introduced interference following a 12-hour, off-line retention period that contained sleep or wakefulness (Fig. 1). Our hypothesis was that if consolidated memories are resistant to interference, and if sleep plays an active role in memory consolidation, then memories would be more resistant to interference after sleep than after similar time periods awake.

### Participants

All potential participants completed a screening questionnaire and interview prior to selection. Individuals taking prescription psychoactive medication or illicit drugs were excluded prior to randomization. All participants were right-handed and native-English speakers. We excluded those with known neurologic, psychiatric or sleep disorders, and those with atypical sleep patterns—i.e., individuals with habitual sleep onset after 2 a.m., sleep duration less then 6-hours. We also excluded those with pathologic sleepiness (defined by an Epworth Sleepiness Scale score >10). Forty-five participants (ages 18–22) were enrolled and successfully completed the study. Prior to the experiment, all participants gave their written informed consent for participation in the research study and for publication of the data. The protocol was approved by the Institutional Review Board at Beth Israel Deaconess Medical Center, an affiliate of Harvard Medical School.

### Materials

Items used in the memory task were two-syllable nouns that were randomly selected from the Toronto Word Pool [14], creating three lists of 60 words. Words in each list were matched for imageability, frequency of use, and concreteness. Word lists were then assigned to two lists of paired associates: A–B and A–C (e.g. BLANKET-VILLAGE and BLANKET-RUBBER). Lists were counterbalanced between participants.

### Procedures

Forty-five participants were randomly assigned to one of two groups: Sleep or Wake. Each participant initially learned 60 paired associates (A–B) in two phases. The first phase of learning was study-only, in which each of the 60 pairs of words, A–B, were presented, one at a time, on a computer screen for 7 seconds each. No specific strategy for encoding was suggested to the participants, other than they should “memorize the words as a pair.” In the second phase of learning, anticipation-plus-study, the computer randomly presented the A words and the participant had to correctly type the answer. Feedback was given in this second phase, i.e., “the correct pairing is….” If the response was incorrect, then that item remained among the randomly displayed items until it was correctly identified. [For details of this training manipulation, see 10], [15]. Some participants were also randomly required to recall the correct word twice before completing the second phase of learning (eight participants in the Sleep group and eight in the Wake group). The remaining participants only needed to correctly recall the B word once in order to complete training. In both instances, the purpose of training was to have the participants learn the A–B pair, such that when presented with any A-word they would correctly recall the associated B-word. And in all cases, the training criterion for learning was 100% accuracy.

Training for the Sleep group took place from 9–10 p.m. and for the Wake group took place from 9–10 a.m.

For testing purposes, the lists of 60 pairs of words were randomly divided into three groups of 20 and used for three tests, each of which were counterbalanced across all subjects. Ten minutes after training, each participant was tested on one of the random set of 20 pairs. The testing method employed cued recall (i.e., presenting the first word, A, and asking the participant to provide its associated B word). This initial test was performed to ensure that there was equal and adequate learning of the items across groups.

Twelve hours after training, participants returned to the laboratory and were tested on the second group of 20 words from the initial 60 pairs, again using cued recall. After a 10-minute break, participants then learned a second word-pair list (A–C) that corresponded to the remaining, untested, 20 A–B pairs. Training of A–C was done in the same manner as training for A–B. After a final 10-minute break, participants were tested on their ability to recall both B and C words of this last 20 cue words (Fig. 1.) In this final test, participants were given the opportunity to answer both the B and C word in order to avoid competition among responses when both the B and C word could be recalled [12]. But the outcome item of interest was the B word. Participants in the Wake group were not restricted from any activity, other then napping, between the training and testing phases of the experiment.

## Results

**Figure 2:**
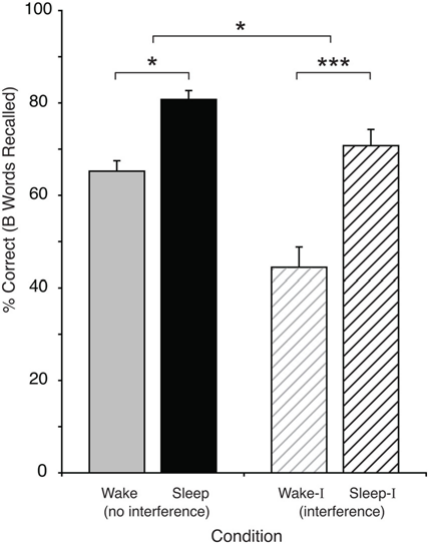
Results. Percent correct recall for B words from the original A–B pair following 12-hour retention interval, with no interference and with interference (A–C) prior to testing. Bar indicates one standard error of the mean. * = *p*<0.05; *** = *p*<0.001.

To test for the effects of Sleep and Interference on recall, we performed a two-way, mixed-effects ANOVA (N = 45) using Sleep v. Wake and Interference v. No-Interference as predictors, and mean recall performance of the B word as the outcome measure. This analysis demonstrated significant main effects of Sleep [F (1,43) = 12.5, *p* = .001] and Interference [F (1,43) = 38, *p* <.0001] on recall accuracy. The Sleep group remembered, on average, 4 more word pairs out of 20. There was also a Sleep-by-Interference interaction [F (1,43) = 4.7, *p* = .036] (Fig. 2), which showed that sleep mitigated the effect of interference, defined as the difference between recall without or with interference. When the amount of training was included in as a covariate in the ANOVA model (either by including trials-to-criterion or performance on immediate recall), the effects of sleep, interference and their interaction remained significant.

We then compared the Sleep and Wake groups to each other in the interference and non-interference conditions (between-group, two-tailed t-tests, assuming unequal variances). In the No-Interference conditions, mean recall was higher in the Sleep group (M = 81%, SD = 18%), than in the Wake group (M = 65%, SD = 22%), *t*(43) = 2.63, *p* = .01. And in the Interference conditions, this difference was even more pronounced—Sleep-Interference (Sleep-I: M = 71%, SD = 21) and Wake-Interference (Wake-I: M = 44%, SD = 24), *t*(43) = 3.96, *p*<.001 (Fig. 2).

The effects of sleep could not be explained by the time of day during which training or testing took place. First, the Wake and Sleep groups were equally able to learn the pairs of words, i.e., neither the number of trials to criterion in learning nor performance on initial testing at 10 minutes were significant (all p>0.2). We also compared second-list recall (C of A–C) at 10-minutes post-training, and found no significant differences between the evening and morning performance: Sleep group (a.m. testing): 95%, SD = 6 and the Wake group (p.m. test): M = 92%, SD = 8; *t*(42) = −1.32, *p* = 0.2).

There was a reliable effect of interference in both the Sleep and Wake conditions. But the effect was twice as large in the Wake group, demonstrating that the negative effect of interference was reduced by sleep: mean effect of interference in sleep = 10.0, SD = 13.2; and mean effect of interference in Wake = 20.8, SD = 18.9, *t*(19) = 3.4, *p* = .003.

## Discussion

Taken together, these data demonstrate that sleep causes recently learned memories to be more accurately recalled than similar time periods of wakefulness. Further, this study reveals that this enormous benefit of sleep is unmasked when using a behavioral paradigm that employs interference directly before testing: sleep had 16% absolute difference when compared to wake in the no-interference conditions, and 27% in the interference conditions. Thus, without using interference testing, we would have underestimated the effect that sleep had on recall performance by nearly half.

This study replicates and extends previous findings by Ellenbogen et al [10]. In that work, as with this study, the large benefit of sleep for memory was optimally revealed when using interference testing. There was, however, a potential problem in the no-interference conditions of that study: the data approached peak performance (i.e., ‘ceiling effect’), thereby limiting our confidence in the effect of sleep in the no-interference comparison, which failed to reach significance, and, by extension, limiting our confidence in the Sleep-by-Interference interaction. The present study demonstrates that when performance is off ceiling, and the study is sufficiently powered, sleep improves recall, even in the absence of interference. Furthermore, when participants are off ceiling, there is still a reliable Sleep-by-Interference interaction. That is, sleep reduces the negative effect of interfering information on memory.

As a consequence, we believe that our refined behavioral paradigm will be particularly useful in providing greater understanding of how sleep might influence memory.

Our finding is consistent with alternative behavioral paradigms that have attempted to deal with effects of unintentional interference by examining differences between sleep and wakefulness across different amounts of sleep and different times of day [16], [17], [18], [19]. Differently, those studies employed longer durations (24–48 hours) between training and testing, or a brief nap. Their manipulations allowed for positioning sleep at different points between training and testing, using time awake as a surrogate for interference. They provide insight toward greater understanding about the nature of the timing of when learning occurred in proximity to sleep. A potential advantage of our paradigm is that it efficiently probes immediate, delayed and post-interference recall from the same participant and across a brief interval, all while experimentally imposing a controlled interference exposure.

An important next step is to characterize how sleep renders memories more robust, particularly when confronted with interference. Does sleep lead to a generally stronger memory trace, which is then resilient to interference, or does sleep confer a specific benefit for recall by inoculating memories from the adverse effects of interference? Distinguishing these alternative explanations might have important implications for understanding memory consolidation and the role of sleep therein.

Another important future direction will be to better understand the biological underpinnings of how sleep influences learning and memory. Recent studies have advanced our understanding of the biological mechanisms through which sleep influences memory. For example, several studies have shown a coordinated replay of memories in rodent hippocampus and sensory neocortex, suggesting that replay is a kind of rehearsal that enhances memory [20], [21] Recent work has made important strides in translating these models into human work by demonstrating similar findings of replay during sleep by exploring neuroimaging [22] and depth electrode recordings [23], [24]. And computational models provide potential mechanisms for how properties of sleep might enhance semantic learning [25]. Taken together, these experiments provide an evolving understanding of the biology of how sleep might influence memory. Yet more experiments are needed in order to bridge these diverse approaches.

With different behavioral approaches in different experiments, it becomes increasingly challenging to develop a unified understanding of how sleep influences memory. Based on the current study's data, we believe that our behavioral paradigm that examines interference might be among the most robust methodological approaches to discern the extent of the effects of sleep on memory. Adding elements of interference testing would, therefore, be an effective tool in advancing understanding of the physiology that accounts for this sleep-enhancing effect of memory recall. As a consequence, we will likely gain clearer insight into the process of memory consolidation, the role of sleep, and their interaction.
